# Characterization of the Loss of SUMO Pathway Function on Cancer Cells and Tumor Proliferation

**DOI:** 10.1371/journal.pone.0123882

**Published:** 2015-04-10

**Authors:** Xingyue He, Jessica Riceberg, Sai M. Pulukuri, Steve Grossman, Vaishali Shinde, Pooja Shah, James E. Brownell, Larry Dick, John Newcomb, Neil Bence

**Affiliations:** 1 Oncology Drug Discovery Unit, Takeda Pharmaceuticals International Co., Cambridge, United States of America; 2 Nurix, Inc. San Francisco, United States of America; Rush University Medical Center, UNITED STATES

## Abstract

SUMOylation is a post-translational ubiquitin-like protein modification pathway that regulates important cellular processes including chromosome structure, kinetochore function, chromosome segregation, nuclear and sub-nuclear organization, transcription and DNA damage repair. There is increasing evidence that the SUMO pathway is dysregulated in cancer, raising the possibility that modulation of this pathway may have therapeutic potential. To investigate the importance of the SUMO pathway in the context of cancer cell proliferation and tumor growth, we applied lentivirus-based short hairpin RNAs (shRNA) to knockdown SUMO pathway genes in human cancer cells. shRNAs for SAE2 and UBC9 reduced SUMO conjugation activity and inhibited proliferation of human cancer cells. To expand upon these observations, we generated doxycycline inducible conditional shRNA cell lines for SAE2 to achieve acute and reversible SAE2 knockdown. Conditional SAE2 knockdown in U2OS and HCT116 cells slowed cell growth *in vitro*, and SAE2 knockdown induced multiple terminal outcomes including apoptosis, endoreduplication and senescence. Multinucleated cells became senescent and stained positive for the senescence marker, SA-β Gal, and displayed elevated levels of p53 and p21. In an attempt to explain these phenotypes, we confirmed that loss of SUMO pathway activity leads to a loss of SUMOylated Topoisomerase IIα and the appearance of chromatin bridges which can impair proper cytokinesis and lead to multinucleation. Furthermore, knockdown of SAE2 induces disruption of PML nuclear bodies which may further promote apoptosis or senescence. In an *in vivo* HCT116 xenograft tumor model, conditional SAE2 knockdown strongly impaired tumor growth. These data demonstrate that the SUMO pathway is required for cancer cell proliferation *in vitro* and tumor growth *in vivo*, implicating the SUMO pathway as a potential cancer therapeutic target.

## Introduction

SUMOylation is an evolutionarily conserved ubiquitin-like protein modification process that regulates a diverse set of biological processes [1, 2]. SUMO (small ubiquitin-related modifier) proteins are ~10 kD ubiquitin-like proteins that are covalently attached to substrate lysine residues via an enzymatic cascade analogous to protein ubiquitination. The human genome encodes four SUMO family proteins: SUMO1–SUMO4. SUMO1–SUMO3 are ubiquitously expressed in all tissue types, whereas SUMO4 is expressed mainly in the kidney, lymph node and spleen [1]. The mature forms of SUMO2 and SUMO3 are 97% identical and only share ~50% sequence homology with SUMO1. SUMO1 is conjugated to target proteins as monomer, while SUMO2 and SUMO3 can form poly SUMO chains as a result of acceptor lysine residues present at the N terminus. The function of SUMO4 is less clear as evidence suggests that it is not processed to a mature, conjugatable form like the other SUMO isoforms [1, 2].

SUMOylation is a dynamic and reversible protein modification. Nascent SUMO precursors are proteolytically processed by SUMO-specific isopeptidases (sentrin-specific proteases; SENPs) to reveal a c-terminal glycine residue. These mature SUMO proteins are activated by the E1 heterodimer SAE1 (AOS1)–SAE2 (SUMO Activating Enzyme 2 or UBA2) in an ATP-dependent reaction that promotes thioester bond formation between the SUMO c-terminal glycine and a cysteine in SAE2. SUMO is then transferred to the catalytic cysteine residue of the sole E2 enzyme UBC9 (also known as UBE2I). Finally, SUMO E3 ligases, including the PIAS family proteins, RanBP2 and Polycomb group member Pc2 [1], facilitate the formation of an isopeptide bond between the SUMO Ubl and a target lysine residue on the substrate. The canonical SUMO-modification site contains the consensus motif ΨKxE (where x refers to any amino acid, Ψ is an aliphatic branched amino acid). SUMOylation is a dynamic process that is ultimately reversed by SUMO proteases, or SENPs, which permit the reuse of the SUMO proteins for subsequent conjugation cycles [2]. Similar to the ubiquitin interacting domains associated with the ubiquitin pathway, SUMO interacting motifs (SIMS) have been identified that promote protein SUMOylation or enhance SUMO dependent protein-protein interactions. Canonical SIMs are generally characterized by a short stretch of hydrophobic residues with a consensus sequence and/or phosphorylated serine residues [3]. The potential biological utility of multiple SUMO Ubls combined with the prevalence of SIMs may help to explain the great diversity of biological processes in which SUMOylation is involved.

The biological consequences of SUMOylation include changes in subcellular localization, altered protein-protein interaction, altered activity and substrate stability [1, 2]. Numerous SUMO targets have been identified through cell and biochemical studies and many of these substrates are involved in key cellular processes and structures, including the cell cycle, chromosome structure and segregation, ribosomal biogenesis, nucleocytoplasmic transport, sub-nuclear structure, DNA repair, and gene expression [4–8]. Several transcription factors, such as KAP1, Sp3, p300, c-Jun and c-Myb, have been reported as SUMO substrates whereby SUMOylation is capable of promoting both transcriptional activation and repression [6, 7, 9]. In addition, SUMO is involved in nuclear transport via the SUMOylation dependent nuclear pore localization of RanGAP1, which enables the maintenance of the RAN GTP gradient that is important for active nuclear import and export [4]. Within the nucleus, SUMOylation is also reported to play important roles in PML nuclear body (NB) formation and recruitment of PML associated proteins including Daxx and SP100 [10, 11].

Of particular interest from a cancer perspective is the substantial evidence that SUMOylation is important for a multitude of processes critical for mitosis. In *S*. *cerevisiae*, cohesin is SUMOylated, and SUMOylation is required for sister chromatid cohesion[12]. The mitotic kinase Aurora B, Topoisomerase IIα, and numerous centromeric and kinetochore proteins are also reported SUMO substrates [13]. Blastocysts isolated from mouse Ubc9 knockout embryos display hypocondensed chromatin and chromosome segregation defects [14]. Similar effects on chromosome segregation have also been observed in genetic studies in *S*. *cerevisiae*, Zebrafish and Drosophila, suggesting an evolutionarily conserved role for SUMO in mitosis. Intriguingly, studies in Drosophila indicate that dividing cells are especially sensitive to the loss of SUMO pathway function relative to non-dividing, differentiated cells [15].

There are emerging connections between protein SUMOylation and cancer. In genome-wide RNAi screens, several SUMO pathway components scored as essential genes for cell proliferation [16]. Both *UBC9* and *SENP1* are required for mouse embryonic development [14, 17]. Moreover, the SUMO E1 enzyme (SAE1/2), was identified as synthetic lethal with c-myc in a genome-wide RNAi screen, and SAE2 is required for growth of Myc-dependent breast cancer in mice [18]. Consistent with this finding, a recent study found loss of SUMOylation induced rapid regression of Myc-driven lymphoma[19]. Elevated levels of UBC9 have been observed in several malignancies and are associated with poorer patient outcome; including lung, colorectal, prostatic, ovarian, breast cancer and melanoma [20–24]. In addition, elevated levels of the SUMO E1, SAE, has also been reported to be associated with worse outcome in breast cancer. These findings warrant the further evaluation of SUMOylation pathway enzymes as potential oncology therapeutic targets.

Validating the potential to find small molecule modulators of the SUMO pathway, inhibitors of SAE and UBC9 [25–28] have been reported although none are currently in clinical development. However, an inhibitor of a related E1 enzyme, the NEDD8 activating enzyme (NAE), is in clinical development [29, 30]. This inhibitor, MLN4924 (pevonedistat), binds to the adenylate binding site of NAE-NEDD8 thioester and utilizes a substrate assisted mechanism of inhibition whereby NAE catalyzes the formation of a NEDD8-MLN4924 adduct that acts as a potent inhibitor of the enzyme. SAE was shown to be capable of forming SUMO compound adducts with a non specific E1 inhibitor (compound 1), demonstrating biochemical proof of concept that SAE could be targeted in this manner[31].

Given the emerging relationship between protein SUMOylation and cancer, we sought to characterize the effects of loss of SUMO pathway function in cancer cell proliferation and tumor growth. We applied stable and conditional shRNA systems to knockdown the SUMO E1 and E2 enzymes, SAE2 and UBC9, in human cancer cell lines and SAE2 in xenograft tumor models. SUMO pathway knockdown resulted in multiple terminal outcomes including senescence and apoptosis, which led to potent proliferation arrest and cell death in cultured cancer cells. To study potential mechanisms, we confirmed the loss of TopoIIα SUMOylation and disruption of PML NBs in HCT116. In addition, our data suggest loss of SUMOylation delayed tumor progression in xenograft models, suggesting SUMO pathway is a potential oncology target.

## Materials and Methods

### Cell Culture and Reagents

HCT116, U2OS and Hela cells were obtained from American Type Culture Collection. HCT-116 and U2OS cells were cultured in McCoy's 5A medium supplemented with heat-inactivated 10% fetal bovine serum. Hela cells were cultured in DMEM medium supplemented with heat-inactivated 10% fetal bovine serum. p300 cells were obtained from Dr. Ron Hay and cultured in DMEM medium supplemented with heat-inactivated 10% fetal bovine serum, 0.5mg/ml G418 (Geneticin) and 0.5mg/ml zeocin.

All the cell lines were infected to express a non-targeting shRNA, SAE2 shRNA or UBC9 shRNA using packaged lentiviral particles (Sigma Mission shRNA library clone#: SAE2 sh1: TRCN0000007470; SAE2 sh2: TRCN0000007472; Ubc9 sh1: TRCN0000007205; Ubc9 sh2: TRCN0000007206; Ubc9 sh3: TRCN0000011077). Infected cells were selected by puromycin for 2 days, left to recover for 24 h and then used for western blot and a variety of growth assays. p300 cells were lysed to measure firefly luciferase activity with Steady-Glo Luciferase Assay System (Promega).

HCT116 and U2OS cells were engineered to contain a vector expressing a Tet-inducible promoter non-targeting shRNA or a hairpin targeting human SAE2. For these cells, the shRNA was a 22-mer stem–loop cloned under the control of a Tet-inducible promoter and shuttled into the pTRIPZ vector (Open Biosystems). The engineered lines were generated by stable transduction with packaged lentiviral particles. Infected cells were selected by puromycin for 2 days, left to recover for 24 h and then treated with 1ug/ml Doxycycline (Sigma). sh1: TATTCCTACTTTCAATACAGCA; sh2: TTCAATTTGGACATCTGGTGCT; sh3: TTGACTTTGTACTTCAGCCCAG; sh4: TTTACATCTAGAACCTGCTGGT.

### Western analysis

Whole cell lysates or tumor extracts were fractionated by non-reducing SDS–PAGE and immunoblotted with antibodies to SAE2 (for cell lysates: polyclonal rabbit antibody generated by Millennium, antibody was validated on cell lines overexpressing SAE2; for tumor extract: epitomics T0083, rabbit), Ubc9 (epitomics 2426–1, rabbit), SUMO1 (epitomics S2227, rabbit), SUMO2/3 (Cell Signaling Technologies 4971, rabbit), RanGAP1 (abcam ab2081, goat), p53 (Cell signaling Technologies 9282, rabbit), p21 (Santa Cruz sc-397, rabbit), cleaved caspase 3 (Cell Signaling Technologies 9661, rabbit), TopoIIα (Cell signaling Technologies 12286, rabbit). All primary antibodies were used 1:1000 dilution. Secondary HRP-labeled antibodies to goat IgG (Millipore, 1:2000 dilution) were used for RanGAP1 primary antibody and blots were developed with ECL reagent (Amersham). For other primary antibodies, the secondary antibody was Alexa-680-labelled antibody to rabbit/mouse IgG (Invitrogen, 1:5000 dilution) and the blots were imaged using the Li-Cor Odyssey Infrared Imaging system. Tubulin (Sigma) was blotted for a loading control.

### Immunofluorescence and nuclear imaging

HCT-116 cells were grown on poly-D-lysine-coated glass coverslips (BD Biosciences) with medium + or—Doxycycline and incubated for 5 days. For PML staining, cells were fixed with 2% paraformaldehyde in PBS for 3 min, permeabilized with 0.5% Triton X-100 in PBS for 10 min, then fixed again with 4% paraformaldehyde in PBS for 5 min and washed in PBS. For Daxx staining, cells were permeabilized with 0.5% Triton X-100 in PBS for 2.5 min first, then fixed with 4% paraformaldehyde in PBS for 10 min and washed in PBS. For immunofluorescence staining, cells were treated with Blocking Reagent (Roche) for 1hr at room temperature and stained with primary antibodies: PML (Santa Cruz sc-966), Daxx (Santa Cruz sc-7152) in Blocking Reagent overnight at 4 degree. Cells were washed with PBS and stained with Alexa 488-conjugated goat anti-mouse IgG (1:2000; Invitrogen) or Alexa 488-conjugated goat anti-rabbit IgG (1:2000; Invitrogen) in Blocking Reagent for 1 hour at room temperature. Cells were washed with PBS, stained with Hoechst 33342 (1:10,000, Invitrogen) for 5 min and washed in PBS. The coverslips were mounted on glass slides with Vectashield (Vector Laboratories). Fluorescently labeled cells were visualized using a Nikon TE 300 fluorescent microscope and images were captured using a digital camera (Hamamatsu).

### Proliferation and cell cycle analysis

After selection, cells were plated in six-well plates (2000 cells/well) with medium + or—Doxycycline and incubated for 12 days. Cells were fixed using 4% para-formaldehyde and then stained with a 0.5% crystal violet solution to identify the presence of cell colonies.

HCT116 cells harboring tet-inducible hairpins were treated with Doxycycline for 6 days and collected cells were fixed in 70% ethanol overnight at 4°C. Fixed cells were centrifuged to remove ethanol, and the pellets were resuspended in propidium iodide and RNase A in PBS for 1h on ice protected from light. Cell-cycle distributions were determined using flow cytometry (FACS Calibur, Becton Dickinson) and analyzed using Winlist software (Verity).

SA-β-Gal staining was conducted using senescence detection kit (abcam) according to the instructions of manufacture.

U2OS Cells treated with Doxycycline 7days were labeled with BrdU for 0.5h, and G1, S and G2/M populations were measured by the BrdU APC flow kit (BD Biosciences). Representative data was shown from independent experiments.

### Tumor xenograft experiments

This study was approved by the Takeda Oncology Company Institutional Animal Care and Use Committee (IACUC) (protocol 09–011). 8 weeks old female NCR nu/nu mice (Taconic Farm Inc.) were used in all in vivo studies. All animals underwent an acclimation period of at least 3 days before they are used for any experimental purposes (4 mice housed per cage). For all procedures performed in this study, animals were anesthetized using an induction of a 4–5% isoflorane/oxygen mixture in a box chamber and then maintained at 1–2% isoflorane/oxygen. Proper animal sedation was confirmed by toe pinching. For the tumor growth progression studies, mice were inoculated with 2 × 10^6^ HCT116 or HT29 cells harboring non-targeting or SAE2 shRNAs subcutaneously in the right flank, and tumor growth was monitored with caliper measurements (for each group, total 4–6 mice were used). When the mean tumor volume reached approximately 200 mm^3^, animals were treated with irradiated 0.0625% Doxycycline Diet (Lab Diet) continuously, which provided 1–6 mg of Dox per mouse/per day. RFP fluorescent imaging was performed on anaesthetized animals using a Xenogen imager before and after Doxycycline diet started. During the study, any animal which met the following criteria were removed from study and euthanized: 1. Weight loss: The body weight of all animals was monitored throughout the study and animals were euthanized if they incurred 15% weight loss between observations. 2. Tumor size: If the tumor volume reached 10% of body weight, tumor size greater than 2cm interferes with eating, drinking, urinating, defecating or walking. 3. Ulceration/necrosis of the tumor site. 4. Paralysis: Loss of function in either forelimbs or hindlimbs. 5. Other criteria that will initiate daily monitoring and euthanasia if not responsive to palliative treatment include: Dehydration, hypothermia, abnormal breathing, low activity level, obvious pain, and general poor body condition. At the end of the study, all animals were euthanized using CO2 asphyxiation followed by thoracotomy to confirm death.

## Results

### Lentivirus based SUMO pathway gene knockdown inhibits cancer cell proliferation *in vitro*

**Fig 1:**
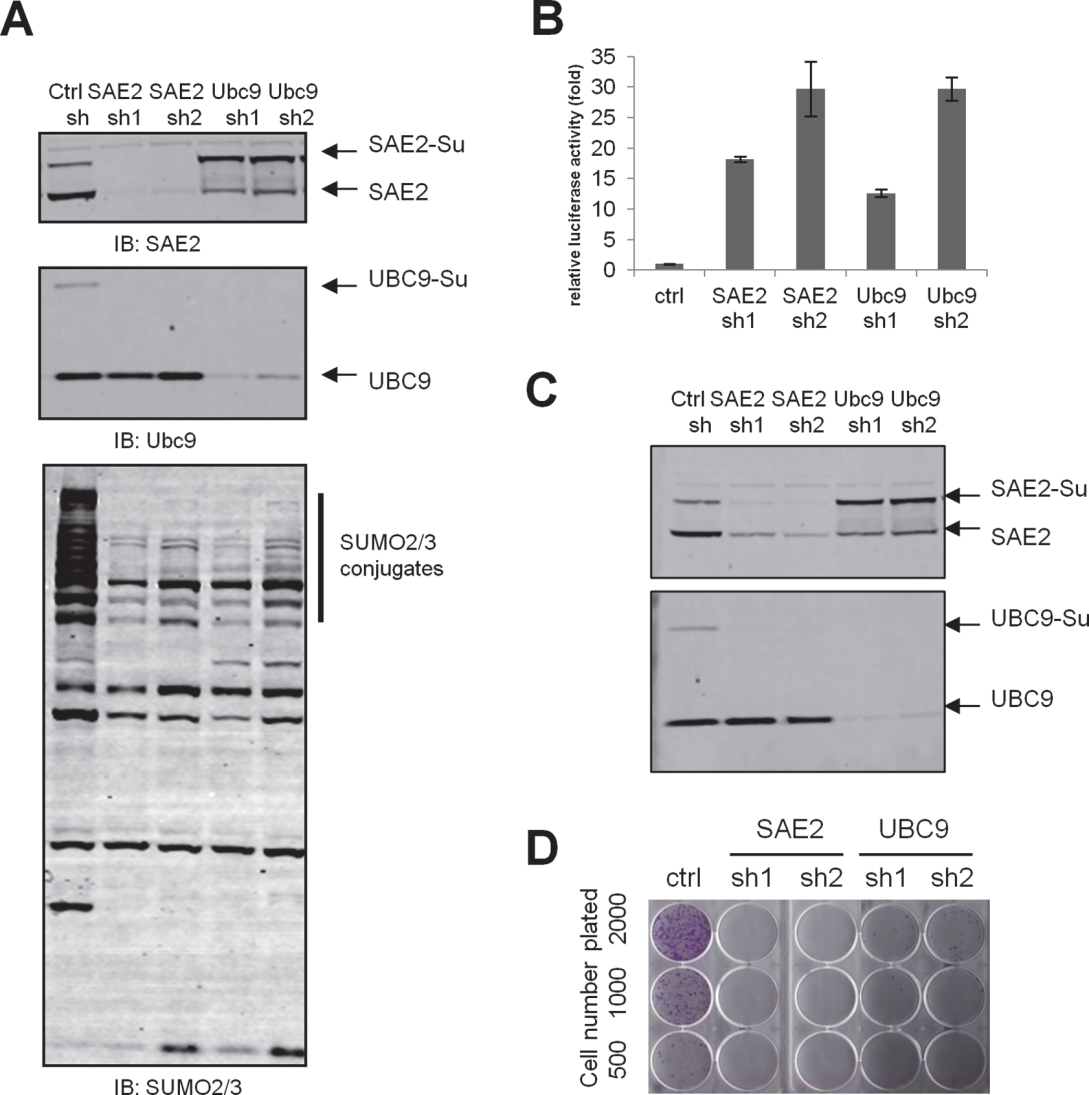
Constitutive SAE knockdown suppresses cell proliferation *in vitro*. (A) SAE2 or UBC9 knockdown reduced SUMO pathway activity. U2OS cells were stably infected with lentivirus expressing shRNAs, selected with puromycin and immunoblotted for indicated proteins. sh1 and sh2 are two shRNAs for each gene. (B) SAE2 or UBC9 knockdown de-repressed a SUMO-repressive reporter. Stable U2OS cells harboring a SUMO-repressive luciferase reporter (p300 cells) were infected with indicated shRNAs. Luciferase activity was measured at Day 5 post puromycin selection. (C) SAE2 or UBC9 knockdown reduced UBC9 thioester level in p300 reporter cells. (D) SUMO inhibition suppressed colony formation. Cells were plated in 6-well plates and stained for crystal violet after 12 days.

To confirm that the SUMO pathway is required for cancer cell survival, we applied a lentivirus based shRNA system to stably knockdown SUMO pathway genes in human cancer cell lines. Two independent shRNAs targeting SAE2 (SUMO E1) or UBC9 (SUMO E2) were infected in U2OS cells (Fig 1A). By immunoblotting, SAE2 shRNAs (sh1 and sh2) efficiently reduced SAE2 protein level and consequently down-regulated UBC9-SUMO thioester levels (Fig 1A), indicating SUMO E1 inhibition functionally impaired SUMO E2 activation. Similarly, two UBC9 shRNA (sh1 and sh2) reduced both the total UBC9 and the UBC9 SUMO thioester levels (Fig 1A). Consistent with the reduced SUMO E1 and E2 levels, we observed reduced levels of SUMO2/3 conjugated proteins (Fig 1A, lower panel), suggesting SAE2 and UBC9 shRNAs are functionally inhibiting SUMOylation in the cells.

To quantitatively measure the SUMOylation inhibition in SAE2 or UBC9 knockdown in cells, we applied a cell-based reporter assay to measure SUMO activity. We harnessed stable U2OS cells with a SUMO repressive luciferase reporter [5]. The cells expressed a GAL4-p300 N-terminus fusion protein (referred as GAL4-p300N thereafter) and harbored a GAL4 binding site in front of the promoter of a luciferase gene. p300 N-terminus contains two known SUMOylation sites. When SUMOylated, GAL4-p300N recruits transcriptional repressors to the GAL4 site which inhibits luciferase transcription. De-SUMOylation of GAL4-p300N de-represses the luciferase reporter. As shown in Fig 1B, SAE2 shRNAs and UBC9 shRNAs increased luciferase signal compared to a control shRNA. Concordantly, SAE2 and UBC9 protein levels were efficiently reduced in these cells (Fig 1C) confirming that SAE2 and UBC9 knockdown functionally reduced SUMOylation activity.

To study the impact of SUMO inhibition on cell proliferation, we plated U2OS cells expressing SAE2 or UBC9 shRNAs and performed a 2D colony formation assay. As shown in Fig 1D, SAE2 and UBC9 shRNAs (sh1 and sh2) potently blocked colony formation *in vitro*. We also measured cell growth by population doubling assay. Consistent with Fig 1D, SAE2 knockdown resulted in slowed cell population doubling (S1A Fig). Similar results were observed in HCT116 and Hela cells (data not shown). These data confirm that SUMOylation is required for cancer cell proliferation.

### Conditional SAE2 knockdown attenuates SUMO pathway activity

**Fig 2:**
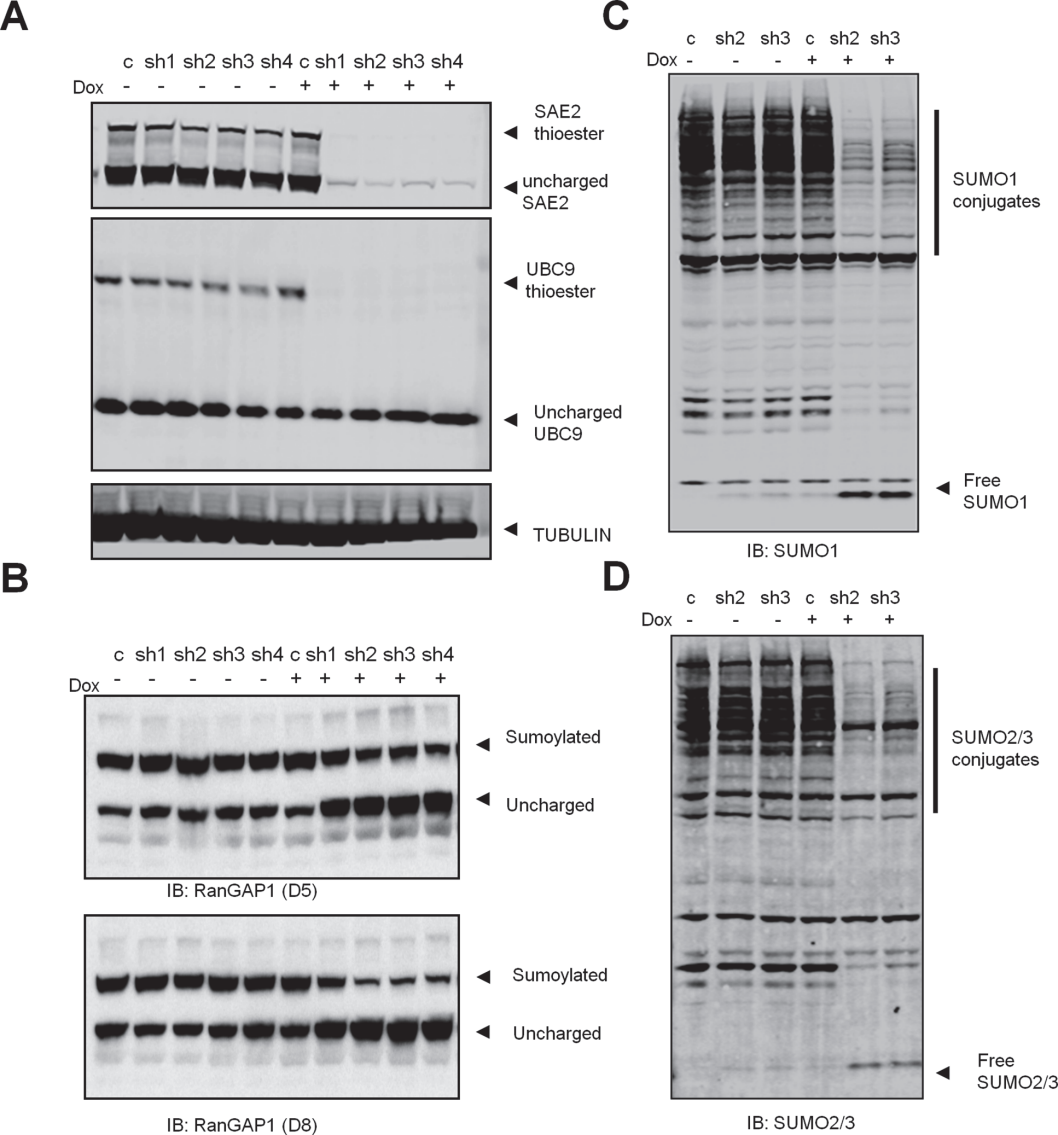
Conditional SUMO knockdown attenuates SUMO pathway activity. (A) U2OS cells were infected with Dox inducible SAE2 shRNAs (sh1-sh4) or control shRNA (c). Cells were treated with Dox for 5 days (+) or untreated (-). Protein lystes were immunoblotted for indicated proteins. TUBULIN serves as loading control. (B-D) U2OS cells expressing conditional SAE2 shRNAs were treated with Dox and immunoblotted with RanGAP1 (B), SUMO1 (C) and SUMO2/3 (D) antibodies.

When growing U2OS cells with SAE2 shRNAs, we noticed that cells which survived after multiple passages showed a much lower SAE2 knockdown compared to early passage cells, indicating that the SUMO shRNAs were negatively selected in this proliferating cell population. To achieve acute and regulatable SAE2 knockdown, we generated four miR30-based SAE2 shRNAs (sh1-sh4) [32] with an RFP (red fluorescent protein) reporter under the control of the TRE promoter. The lentiviral vector also has an rtTA transactivator to form a tet-on shRNA system that is inducible with doxycycline (Dox) [32]. U2OS cells were infected with the conditional SAE2 shRNAs and treated with Dox for 5 days. As shown in Fig 2A (upper panel), upon Dox treatment, conditional SAE2 shRNAs efficiently reduced SAE2 and SAE2-thioster levels whereas a control shRNA (c) did not affect level of uncharged and thioester SAE2. By immunoblotting for UBC9, we showed that conditional SAE2 knockdown reduced levels of the UBC9-SUMO thioesters (Fig 2A, middle panel). Through monitoring the RFP reporter co-expressed from the TRE promoter, we detected RFP induction upon Dox treatment for 36 hrs (S2 Fig), indicating efficient induction of the tet-on shRNA system.

To study whether conditional SAE2 shRNAs suppressed SUMOylation in cells, we measured total SUMO conjugates as well as the known SUMO target gene RanGAP1 [4] in the SAE2 knockdown cell. Both SUMO1 and SUMO2 conjugates were significantly decreased in SAE2 knockdown cells at day 5 with Dox treatment (Fig 2C and 2D, +Dox lanes, sh2 and sh3). In addition, upon acute SAE2 knockdown, the level of SUMOylated RanGAP1 was decreased at 5 days post Dox treatment and the reduction peaked at day 8 (Fig 2B). This observation is consistent with reported prolonged half life of SUMO modified RanGAP1 [33]. We also tested the conditional shRNA system in HCT116 cells and observed similar acute knockdown of SAE2 and accompanied SUMOylation inhibition (S3 Fig). Our results showed that conditional shRNAs can acutely knock down SAE2 to attenuate SUMO pathway activity in cancer cells.

### Conditional SAE knockdown induced apoptosis, endoreduplication and senescence

**Fig 3:**
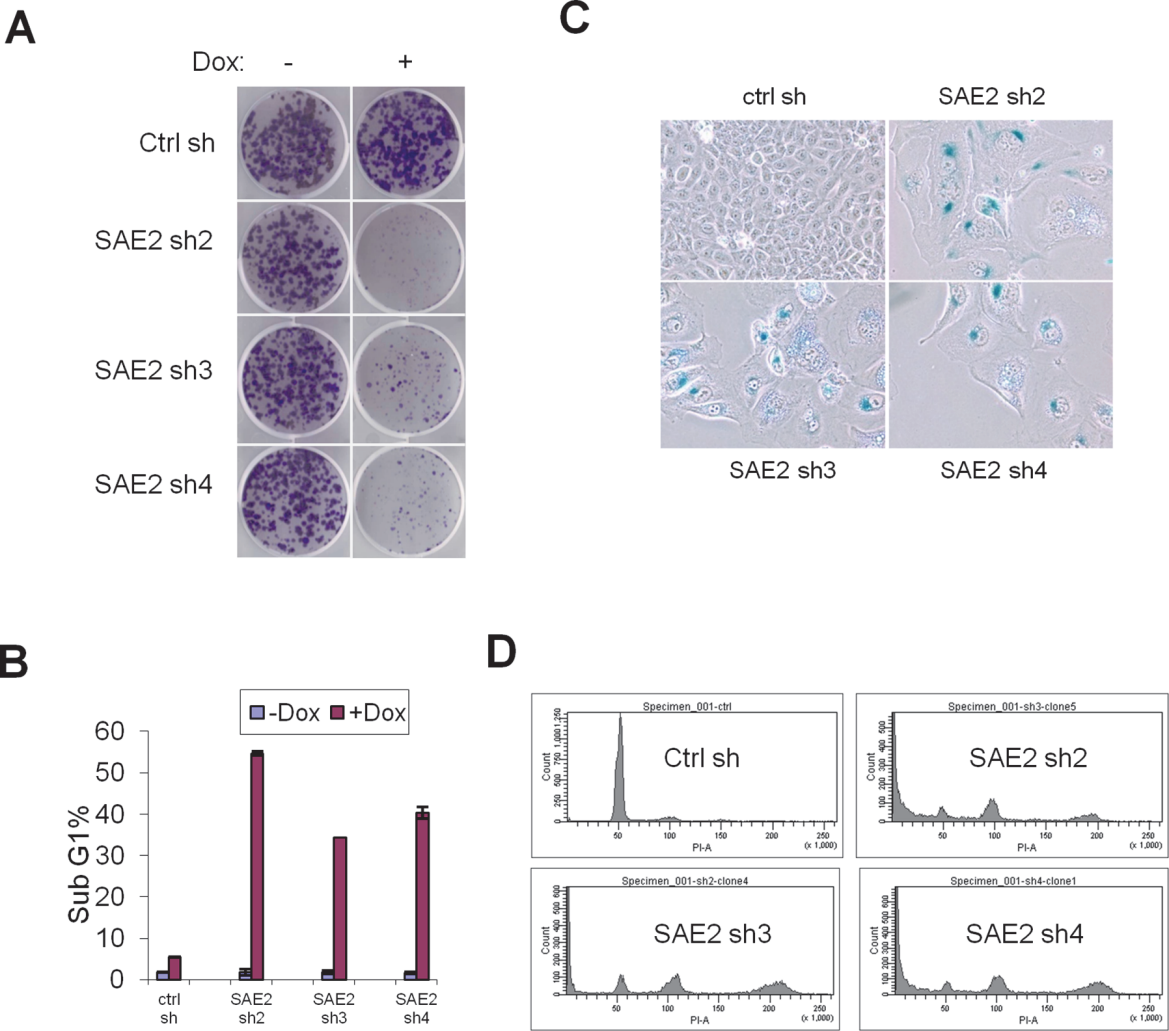
Conditional SAE knockdown induces cell cycle arrest, apoptosis and senescence. (A) HCT116 cells were infected with Dox inducible SAE2 shRNAs or control shRNA (ctrl). Cells were plated in 6-well plates in Dox containing medium (0.5ug/ml) and stained for crystal violet after 12 days. (B) Knockdown of SAE induces apoptosis. Sub G1 population was analyzed by FACS with and without Dox treatment. Error bars denote s.d. (C) SAE2 knockdown induced cellular senescence. Cells were stained for SA-β-Gal activity after 9 days Dox treatment. (D) PI staining and FACS analysis of HCT116 cells following SAE2 knockdown. Sub G1 and mutil-nucleated cell population was indicated.

To address how acute SAE2 knockdown impacts cell proliferation *in vitro*, we performed a colony formation assay in Dox treated HCT116 and U2OS cells expressing conditional SAE2 shRNAs (Fig 3A). Consistent with stable knockdown, Dox treatment significantly blocked colony formation in three SAE2 shRNAs groups (sh2-sh4) but not in the control shRNA group (Fig 3A and S4A Fig).

To explore the potential phenotypes that promote SAE2 knockdown-mediated growth arrest, we assayed apoptosis and cell cycle markers in SAE2 knockdown cells. By PI staining and flow cytometry, we observed increased sub-G1 cell population in HCT116 cells upon conditional SAE2 knockdown (Fig 3B), suggesting some cells are undergoing apoptosis. By BrdU incorporation assay, we observed a decreased percentage of cells in S phase in SAE2 knockdown U2OS cells (S4B Fig). In addition to apoptosis and reduced cell growth, starting from 6 days post continuous Dox treatment, SAE2 knockdown HCT116 cells also showed a multi-nucleated phenotype with enlarged and flattened morphology which is commonly observed in senescent cells. This cell population stained positive for SA-β-Gal activity (Fig 3C), indicating that these cells have initiated a senescence program. Moreover, flow cytometric analysis of nuclear DNA content in Dox treated cells showed increased 8N DNA content from HCT116 cells harboring SAE2 shRNAs, suggesting that impaired SUMOylation leads to endoreduplication (Fig 3D). These results suggest that reduced SUMOylation leads to a mitotic defect impaired cytokinesis, which likely contributes to observed apoptosis and senescence.

### SAE2 shRNA phenotype is rescued by a non-silencible cDNA

**Fig 4:**
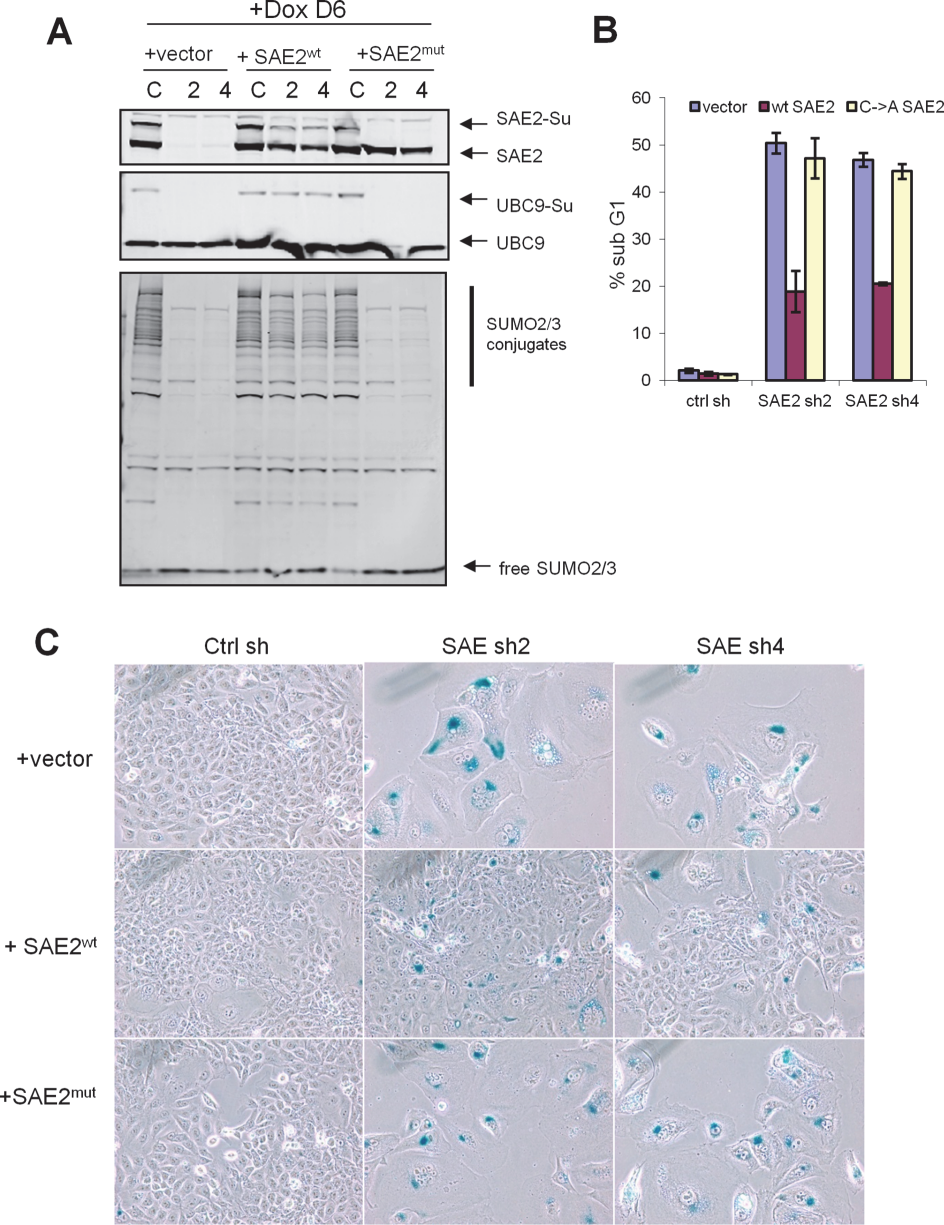
SAE2 shRNA is rescued by non-silencible cDNA. (A) HCT116 cells expressing tet-on inducible shSAE2 (C = ctrl shRNA, 2 = sh2, 4 = sh4) were infected with vector, non-silencible wildtype SAE2 or non-silencible C->A enzyme dead SAE2 mutant. Cells were treated with Dox for 6 days. Protein lysates were immunoblotted with indicated antibodies. (B) non-silencible wildtype SAE2 rescues shSAE2 induced cell death. Sub G1 population was analyzed by FACS. Error bars denote s.d. (C) SA- β-Gal staining of cells expressing indicated retrovirus combination.

To rule out the off-target effects of RNAi, we performed a rescue experiment with siRNA refractory cDNA constructs. Cells expressing tet-on shSAE2 were infected with empty vector, non-silencible wildtype SAE2 (SAE2^wt^), or a non-silencible inactive SAE2 C173A mutant cDNA (SAE2^mut^). Without Dox treatment, the SUMO conjugate levels are similar within these infected cell lines, examined by western blot (S5 Fig). After 6 days continuous Dox treatment, compared to vector control, SAE2^wt^ partially rescued down-regulation of SAE2-SUMO and UBC9-SUMO thioester levels and partially restored SUMO2/3 conjugates in shSAE2 expressing cells. In contrast, the expressed inactive SAE2^mut^ failed to rescue the SAE2-SUMO thioester, the Ubc9-SUMO thioester and SUMO conjugates (Fig 4A). As further confirmation that the non-silencible SAE2 can functionally rescue the SUMO pathway activity, we performed the same sub-G1 apoptosis assay and SA-β-Gal staining. The wildtype SAE2, but not the C->A enzyme dead SAE2 mutant, partially rescued shSAE2 induced multinucleation and senescence, and associated cell death (Fig 4B and 4C). These data support that the mitotic defects and apoptosis phenotypes of SAE2 shRNA are likely caused by on target impairment of SUMO pathway activity.

### Inhibition of SUMO pathway leads to DNA decatenation defect and PML NB disruption

**Fig 5:**
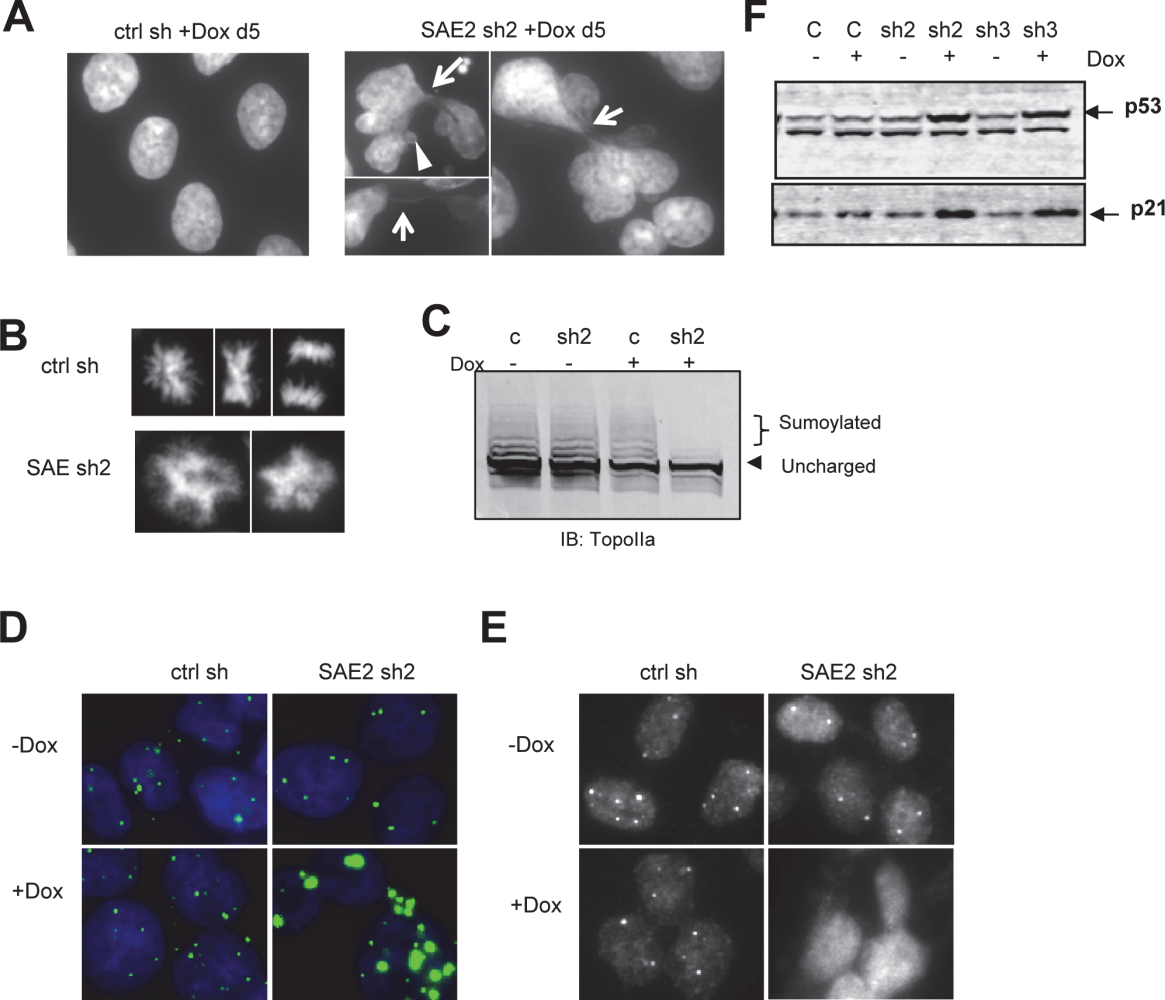
Conditional SAE knockdown induces DNA bridges, mitotic defect and PML NB disorder. (A-B) HCT116 cells expressing tet inducible SAE2 shRNA (sh2) or control shRNA (ctrl) were treated with Dox containing medium (0.5ug/ml) for 5 days. The cells were fixed and stained with DAPI to view nuclei morphology. (C) HCT116 cells expressing tet inducible SAE2 shRNA (sh2) or control shRNA (ctrl) were treated with Dox for 4 days then immunoblotted with TopoIIα antibody. (D-E) immunofluorescence images of HCT116 cells harboring inducible shRNA with 5 days treatment of Dox. The cells were stained with (D) PML or (E) Daxx antibodies. (F) SAE2 knockdown is associated with increased p53 levels. U2OS cells expressing conditional SAE2 shRNAs were treated with Dox and immunoblotted with p53 and p21 antibodies.

A key question is how loss of SAE2 activity leads to endoreduplication and apoptosis. After 5 days of continuous treatment with doxycycline, cells expressing SAE2 shRNAs developed abnormal nuclear morphology (Fig 5A). Cells frequently developed enlarged nuclei and commonly displayed multi-lobed nuclei (Fig 5A), thin DNA bridges connecting two nuclei (Fig 5A, arrow), and micronuclei (Fig 5A, arrowhead). Mitotic abnormalities including multipolar spindles were also observed (Fig 5B). These results recall the phenotype observed with defect in DNA decatenation, where the segregation failure of entangled chromosomes can generate DNA bridges which lead to abnormal mitosis, impaired cytokinesis and can reduce proliferation and viability [34]. Previous studies have reported that Topoisomerase IIα (TopoIIα) is a SUMO substrate and SUMOylation is important for TopoIIα activity in vitro [35–37]. Western blots in HCT116 cells expressing control or SAE2 shRNA revealed endogenously SUMOylated TopoIIα species, and knockdown of SAE2 inhibited TopoIIα SUMOylation (Fig 5C).

Since SUMOylation is involved in many cellular processes, and we observed evidence of both apoptosis and senescence, we investigated the effects of SUMO pathway impairment on PML nuclear bodies. PML nuclear bodies are nuclear matrix organizers which regulate key processes such as apoptosis and senescence. In a published report, Ubc9 knockout blastocysts display disrupted PML nuclear bodies and re-distribution of PML associated protein Daxx[14]. We performed immunofluorescence to exam the PML organization in the cells harboring control or SAE2 shRNA. Knockdown of SAE2 led to enlarged PML nuclear bodies and increased PML intensity (Fig 5D). Dissociation of Daxx from PML nuclear bodies was also observed (Fig 5E). Many regulatory factors, such as p53, can be recruited to PML nuclear bodies for post-translational modification[10]. In p53 proficient U2OS cells, we detected induction of p53 and its target gene p21 in Dox treated shSAE2 cells (Fig 5F). Together, these results suggest that impairment of the SUMO pathway is impacting proliferation and viability at multiple biological nodes including DNA decatenation, mitosis and PML nuclear body organization, which in sum likely contribute to the proliferation defects observed in the SAE2 knockdown cancer cells.

### Conditional SAE knockdown impairs tumor growth progression

**Fig 6:**
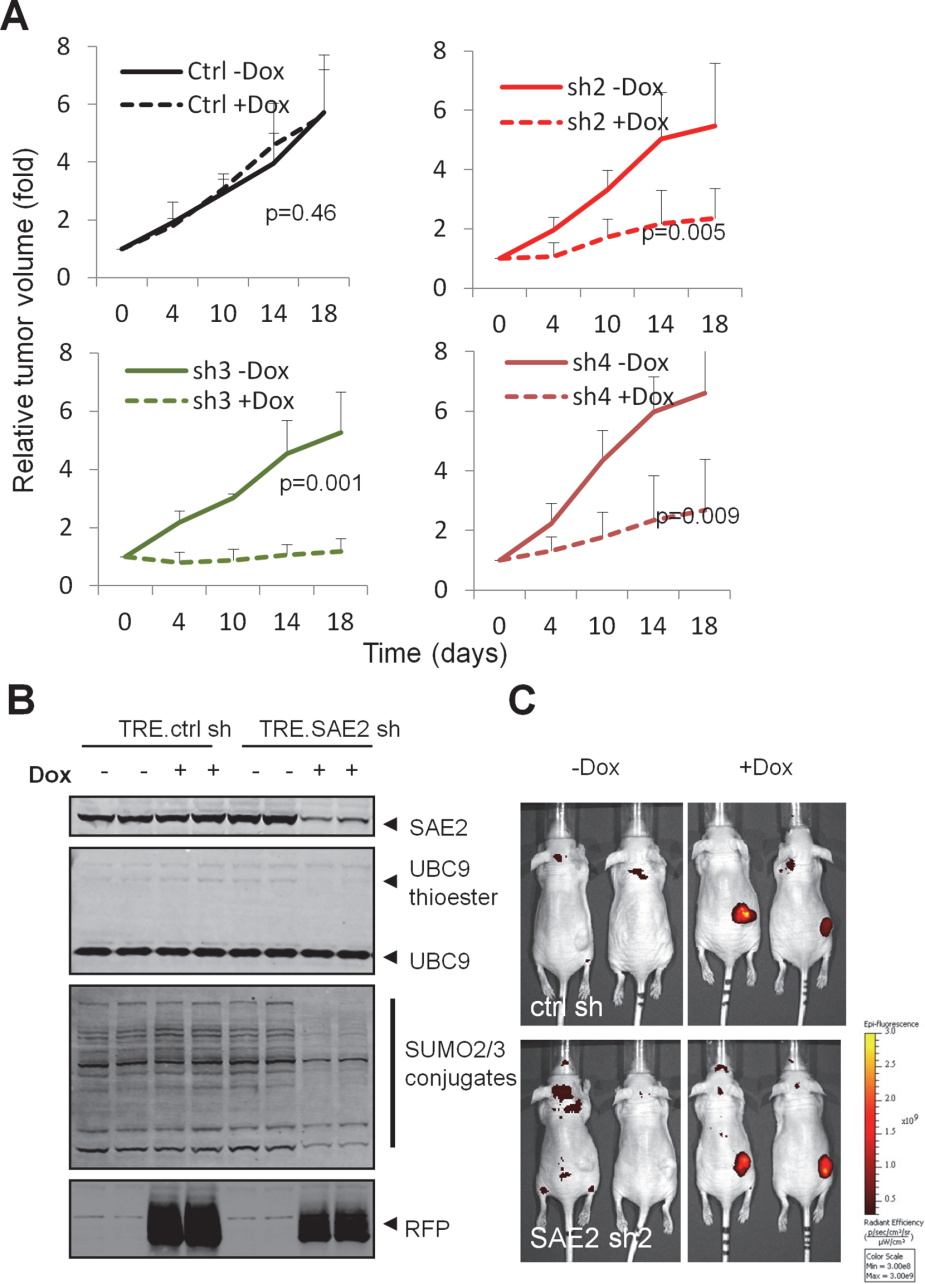
Conditional SAE knockdown delays tumor progression. (A) HCT116 cells infected with Dox inducible SAE2 shRNAs were injected subcutaneously in immunocompromised mice. Dox treatment starts at D14 after injection (set as D0). Error bars denote s.d. (n = 4). (B) SAE2 shRNA reduced SUMO pathway activity in tumors. Protein lysates from tumors were immunoblotted with indicated antibodies. (C) Whole-body fluorescent imaging of mice untreated or treated with Dox. Color bar denotes signal intensity.

To address whether acute SAE2 knockdown can influence cancer cell survival *in vivo*, we applied the HCT116 xenograft tumor model [29]. We transplanted HCT116 cells with control shRNA or SAE2 shRNAs in immunocompromised nude mice, waited 14 days to allow tumors to reach 200mm^3^ and then treated mice with Dox-containing food to induce SAE2 knockdown in the established tumors. As shown in Fig 6A, Dox treatment did not affect the progression of control shRNA tumors (p = 0.46, student t test). In all three SAE2 shRNA tumors, Dox treatment significantly delayed tumor growth.

By measuring SAE2 proteins in tumor lysates, we observed that Dox treated SAE2 shRNA tumors have a decreased level of SAE2, associated with lower levels of UBC9-SUMO thioester and SUMO2/3 conjugates (Fig 6B), suggesting a functional SUMO inhibition *in vivo* by our approach. Because RFP is a reporter co-expressed from the TRE promoter, the induction of RFP in tumors confirmed Dox dependent induction of control and SAE2 shRNAs in tumor (Fig 6C).

We also tested tet-on shSAE2 in a second xenograft tumor model, HT29, with cells expressing tet-on control shRNA or two SAE2 shRNAs. Like in the HCT116 model, tet-on shSAE2 successfully inhibited SUMOylation level *in vitro*, and significantly delayed HT29 tumor growth *in vivo* (S6 Fig). These data confirm that SAE2 knockdown mediated loss of SUMO pathway function induces tumor growth arrest.

## Discussion

SUMOylation regulates a diverse array of biological pathways that are important for many cancer relevant processes [1, 38]. In this study, we applied both constitutive and Dox inducible shRNAs to efficiently knockdown the SUMO E1 and E2 enzymes, SAE2 and UBC9 in human cancer cell lines and xenograft models. We used multiple shRNAs and unsilencible cDNA rescue approach to rule out the off-target effect of RNAi. In our studies, we showed that SUMO E1 and E2 shRNAs reduced SUMO pathway activity measured by cell based reporter assay and SUMO conjugate immunoblots. In cultured human cancer cells, knockdown of SUMO enzymes efficiently suppressed cell growth. Using conditional shRNAs targeting SAE2, we showed that acute SAE2 knockdown leads to a cell proliferation defect, increased apoptosis and marked growth arrest in xenograft tumors. These results demonstrated SUMO pathway is required for the survival and maintenance of tumor cells *in vitro* and *in vivo*.

Our studies demonstrate that acute SAE2 knockdown in human cancer cells induces mitotic defects, endoreduplication and multinucleation leading to cellular senescence and apoptosis. The phenotypes of SAE2 knockdown underscore the potential importance of SUMOylation for a variety of biological processes [1, 2, 6, 11, 38, 39]. It is intriguing to note, however, that the phenotypes recall those observed in a human cell line (HTETOP) with conditional disruption of TopoIIα expression. These cells display enlarged nuclei, endoreduplication and DNA bridges after silencing of TopoIIα[40]. Additionally, catalytic inhibitors of TOPOII such as ICRF-193 which prevent proper DNA decatenation (not to be confused with re-ligation inhibitors like etoposide) have also been shown to induce chromatin bridges and multinucleation [41, 42]. Thus it is plausible that our findings are consistent with previous reports of the role of SUMOylation in regulating TopoIIα function [35, 36]. We observe endogenously SUMOylated TopoIIα in our cancer cell line models, and knockdown of SAE2 specifically inhibited TopoIIα SUMOylation. These data suggest that a DNA decatenation defect may help to explain many of the phenotypes that we observe in our knockdown studies. However, since SUMOylation regulates many cellular pathways, TopoIIα is likely among many SUMO targets mediating the effects. The critical role of SUMOylation in mitosis may also contribute to the observed chromatin bridge, multinucleation and endoreduplication phenotypes. In published reports, *Ubc9* mutant *S*. *cereviseae* cells display defects in anaphase promoting complex/cyclosome (APC/C) activation and subsequent cyclin B degradation[43]. SUMOylation is also reported to be essential for many aspects of mitosis. SUMOylated RanGAP1 forms complex with UBC9 and RanBP2 to bind to outer kinetochores [39] and a large number of mitotic chromosomal structural proteins, such as CENP-E, BubR1, condensins and cohesin are identified SUMO substrates [39]. SUMOylation also may modulate the function of the Aurora-B kinase which plays important roles in ensuring proper chromosomal segregation [44, 45]. Lastly, the BLM helicase is reported to be SUMOylated [46]. BLM helicase was reported to localize with a class of ultrafine anaphase bridges and is required for chromosome segregation [47]. It is therefore possible that the observed mitotic defects and the subsequent development of chromatin bridges, polyploidy, and multinucleation result not only from impaired TopoIIα function but also the cumulative impairment of a significant array of SUMO dependent mitotic events.

It is also important to acknowledge the potential contribution of other SUMO dependent cellular events that could impact proliferation and/or tumor growth. A number of SUMO targets are known to be involved in many other pathways including gene transcription, DNA damage repair, nuclear transport and sub nuclear structure. A recent publication discovered SUMOylation of TFAP2A blocks its transcriptional activity to induce the expression of luminal genes. This study suggested SUMOylation of the TFAP2A transcription factor is critical to maintain the basal breast cancer phenotype, providing therapeutic potential for basal breast cancer[48]. In addition to gene transcription, SUMOylation also reported to play important roles in nuclear structure. Corroborating published findings in the Ubc9 knockout blastocysts [14], we too observed perturbation of the PML nuclear bodies and a re-distribution of the PML associated protein Daxx in cancer cell lines. PML nuclear bodies are reported to play diverse roles in the DNA damage response, angiogenesis, telomere maintenance, apoptosis and senescence. There is evidence to suggest that PML nuclear bodies recruit diverse transcription factors, including E2F and p53, and process post transcriptional modifications to regulate their activities [10]. PML was one of the first identified SUMO substrates and SUMO has been reported as a critical component for PML nuclear body formation and regulation. In addition, a recent study suggests that PML interacts with and SUMOylates misfolded proteins, leading to proteasome mediated protein degradation[49]. Interestingly, we observe that the tumor suppressor p53 (Fig 5F), a key regulator of cell cycle arrest and apoptosis, accumulates upon prolonged SAE2 knockdown. Future experiments will explore how the global deSUMOylation directly or indirectly triggers these tumor suppressive mechanisms.

The *MYC* oncogene is a frequently amplified in human cancer and a recent synthetic lethal shRNA screen identified SAE1 and SAE2 (the two heterodimer components of the SUMO E1) as top hits that confer synthetic lethality in breast cancer cells with high MYC activity [18]. In this study, loss of SAE2 led to apoptosis, mitotic spindle defects, and reduced tumor growth in MYC-dependent breast cancer cells. A switch of a MYC-regulated transcriptional program was also reported. Importantly, MYC-high breast cancer patients with high SAE2 levels showed poor prognosis compared to those with low SAE2, suggesting that elevated SUMO pathway function may promote the tumorigenic state. Consistent with these findings, a recent study found MYC activates gene expression of SUMO pathway components in MYC driven lymphoma, and inhibition of SUMOylation impaired development and maintenance of MYC-driven lymphoma [19]. Moreover, data presented in this study provides proof-of-concept that SUMO pathway inhibition at the levels of E1 (SAE2) and E2 enzymes (UBC9) is sufficient to compromise cancer cell survival beyond breast cancer and lymphoma. The fact that conditional SAE2 knockdown induced general mitotic defects and significantly delayed HCT116 and HT29 xenograft tumor growth (Fig 6 and S6 Fig) indicate SUMO pathway inhibition as a potential therapeutic approach in cancer. Our findings also indicate that anti-proliferative effects of SAE inhibition may extend beyond MYC amplification. Future work will address whether SUMO inhibition affects normal cell growth and whether tumors with certain mutations are selectively sensitive to SUMO inhibition.

In summary, we have shown that the SUMO pathway is required for cancer cell survival and tumor progression. We believe that the primary mediators of these effects are through the disruption of proper chromosome decatenation and segregation. This ultimately impairs functional mitosis and promotes endoreduplication and multinucleation. These cellular insults eventually result in apoptosis and/or a proliferation defect, i.e. senescence, depending on the extent of post mitotic DNA damage that the cell has endured. These *in vitro* and *in vivo* models of conditional SAE2 knockdown may further facilitate the discovery of novel SUMO pathway biomarkers and enable additional studies to more precisely determine the exact SUMO dependent cellular pathways that promote cancer cell growth and tumor maintenance.

## Supporting Information

S1 Fig SAE knockdown suppresses cell proliferation in vitro. Population doubling assay in U2OS cells infected with indicated shRNAs.
(PDF)

S2 Fig RFP reports the Dox induced shRNA expression. U2OS cells were infected with Dox inducible SAE2 shRNAs or empty vector control (ctrl). Cells were imaged 36 hrs after Dox treatment.
(PDF)

S3 Fig Conditional SUMO knockdown attenuates SUMO pathway activity. HCT116 cells were infected with Dox inducible SAE2 shRNAs or control shRNA (ctrl). Cells were treated with Dox for 5 days (+) or untreated (-). Protein lystes were immunoblotted for SAE2, Ubc9 (A), and SUMO2/3 (B).
(PDF)

S4 Fig Conditional SUMO knockdown leads to reduced cell proliferation in U2OS cells. (A) U2OS cells were infected with Dox inducible SAE2 shRNAs or empty vector control (ctrl). Cells were plated in 6-well plates and stained for crystal violet after 14 days. (B) SAE2 knockdown decreased S phase in U2OS cells measured by BrdU incorporation. (C) SAE2 knockdown induced growth arrest in U2OS cells measured by cell population doubling assay. Representative data was shown from independent experiments.
(PDF)

S5 Fig SAE2 shRNA is rescued by non-silencible cDNA. Cells expressing tet-on inducible shSAE2 (C = ctrl shRNA, 2 = sh2, 4 = sh4) were infected with vector, non-silencible wildtype SAE2 or non-silencible C->A enzyme dead SAE2 mutant. Cells were untreated with Dox. Protein lysates were immunoblotted with indicated antibodies.
(PDF)

S6 Fig HT29 cells harboring tet-on SAE2 shRNA result in delayed tumor growth *in vivo*. (A) HT29 cells were infected with Dox inducible SAE2 shRNAs (sh2 and sh4) or empty vector control (ctrl). Cells were treated with Dox for 5 days (+ Dox) or untreated (- Dox). Protein lystes were immunoblotted for SAE2, Ubc9, and SUMO2/3. (B) HT29 cells harboring tet-on ctrl sh or SAE2 sh2 and SAE2 sh4 were injected subcutaneously in immunocompromised mice. Dox treatment starts at D14 after injection (set as D0).
(PDF)
