# Supplementary figures and images for: Characterization of the Loss of SUMO Pathway Function on Cancer Cells and Tumor Proliferation
Source: PLoS One. 2015 Apr 10;10(4):e0123882. doi: 10.1371/journal.pone.0123882 (PMC4393225; doi:10.1371/journal.pone.0123882)

Fig. S1

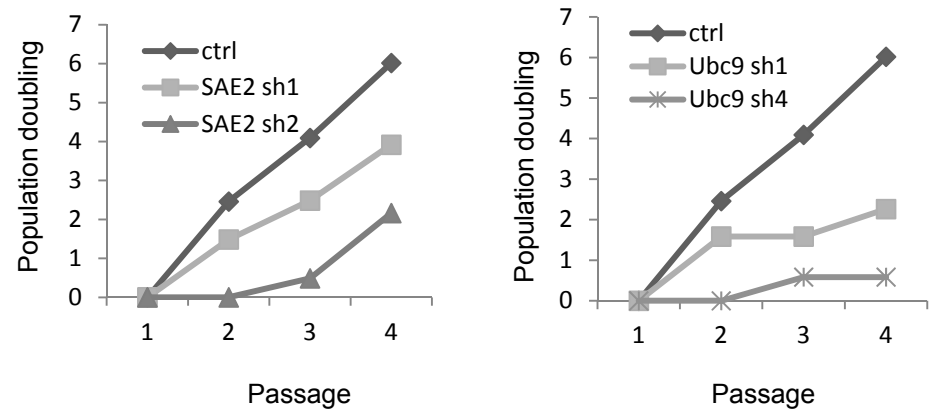

Supplement: S1 Fig — Population doubling assay in U2OS cells infected with indicated shRNAs. (PDF) [file pone.0123882.s001.pdf]

**Fig. S2**

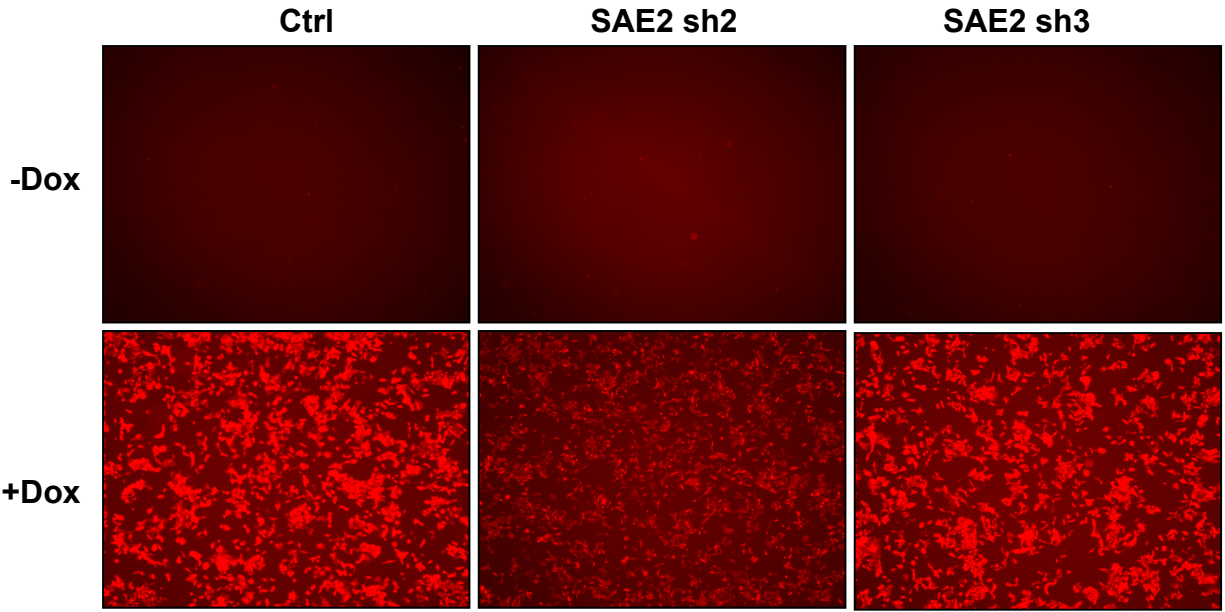

Supplement: S2 Fig — U2OS cells were infected with Dox inducible SAE2 shRNAs or empty vector control (ctrl). Cells were imaged 36 hrs after Dox treatment. (PDF) [file pone.0123882.s002.pdf]

**Fig. S3**

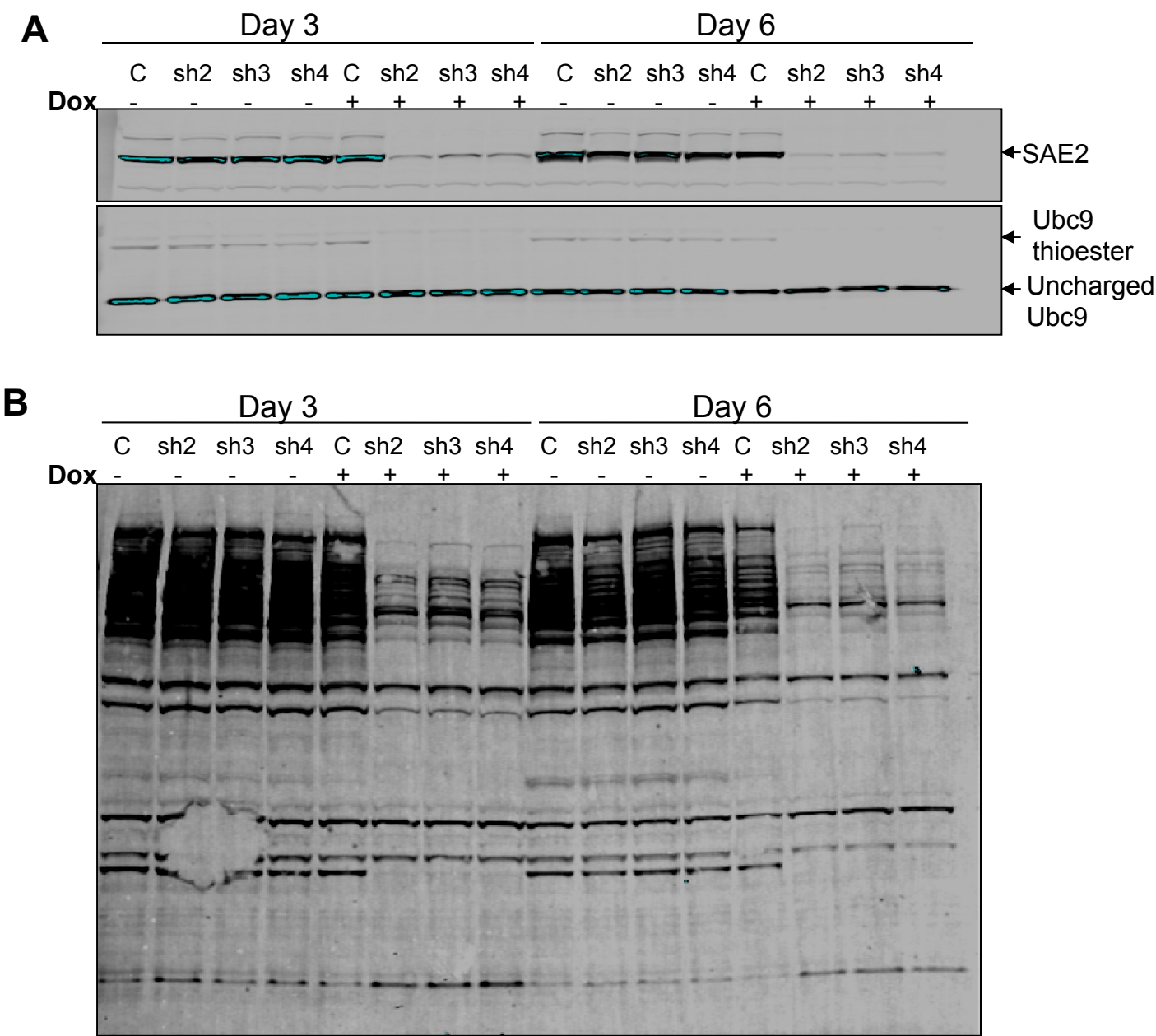

Supplement: S3 Fig — HCT116 cells were infected with Dox inducible SAE2 shRNAs or control shRNA (ctrl). Cells were treated with Dox for 5 days (+) or untreated (-). Protein lystes were immunoblotted for SAE2, Ubc9 (A), and SUMO2/3 (B). (PDF) [file pone.0123882.s003.pdf]

**Fig. S4**

**A**

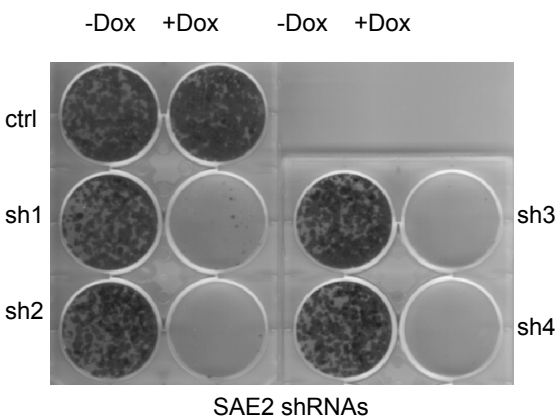

**B**

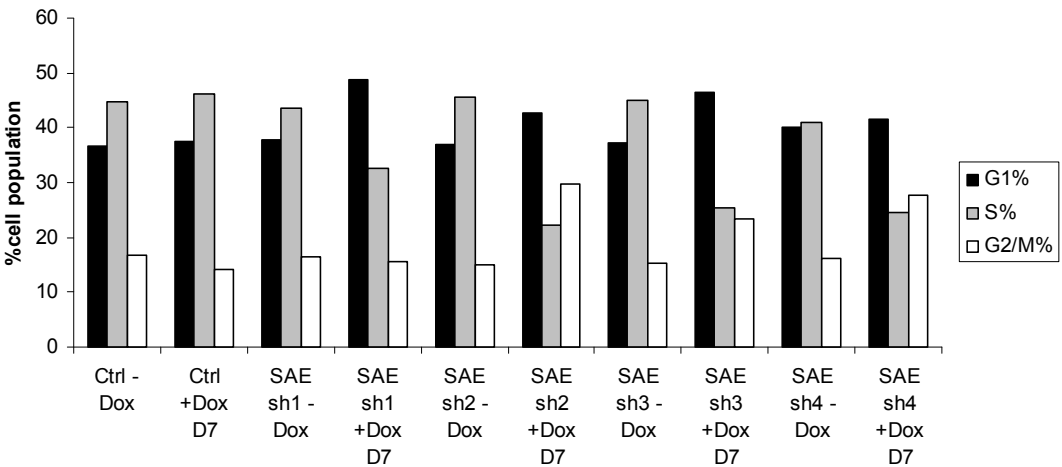

**C**

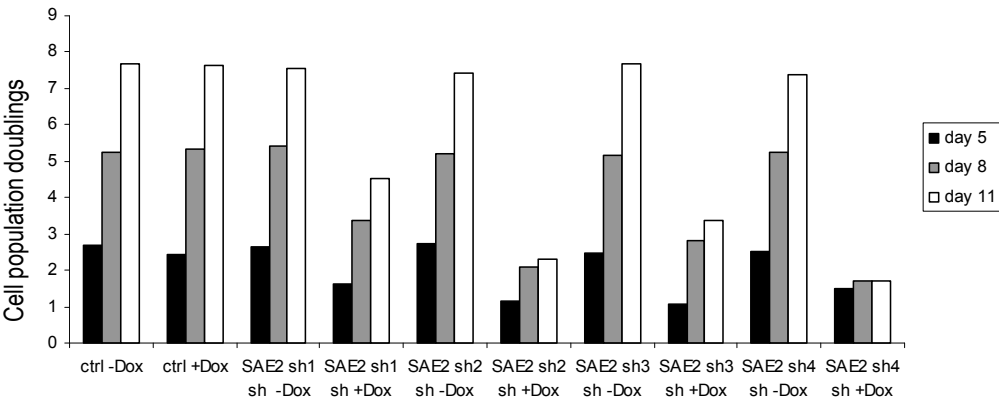

Supplement: S4 Fig — (A) U2OS cells were infected with Dox inducible SAE2 shRNAs or empty vector control (ctrl). Cells were plated in 6-well plates and stained for crystal violet after 14 days. (B) SAE2 knockdown decreased S phase in U2OS cells measured by BrdU incorporation. (C) SAE2 knockdown induced growth arrest in U2OS cells measured by cell population doubling assay. Representative data was shown from independent experiments. (PDF) [file pone.0123882.s004.pdf]

**Fig. S5**

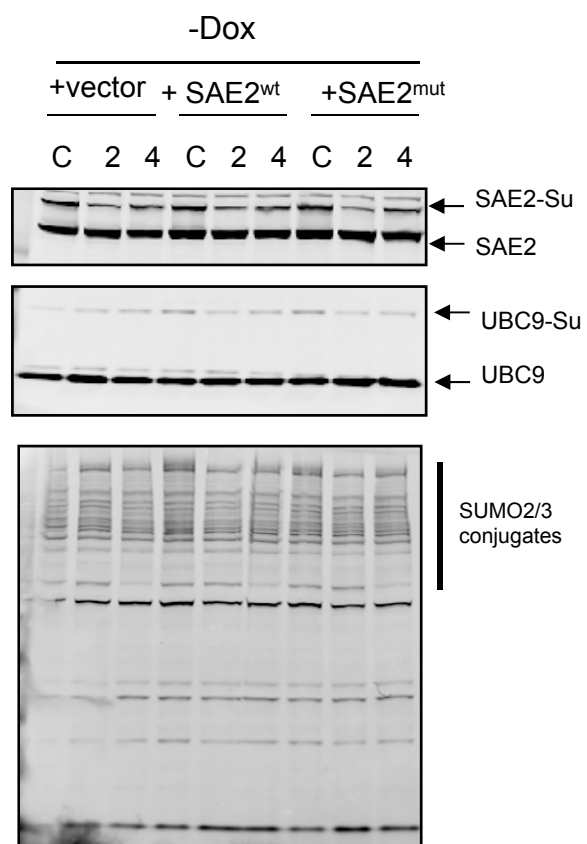

Supplement: S5 Fig — Cells expressing tet-on inducible shSAE2 (C = ctrl shRNA, 2 = sh2, 4 = sh4) were infected with vector, non-silencible wildtype SAE2 or non-silencible C->A enzyme dead SAE2 mutant. Cells were untreated with Dox. Protein lysates were immunoblotted with indicated antibodies. (PDF) [file pone.0123882.s005.pdf]

**Fig. S6**

**A**

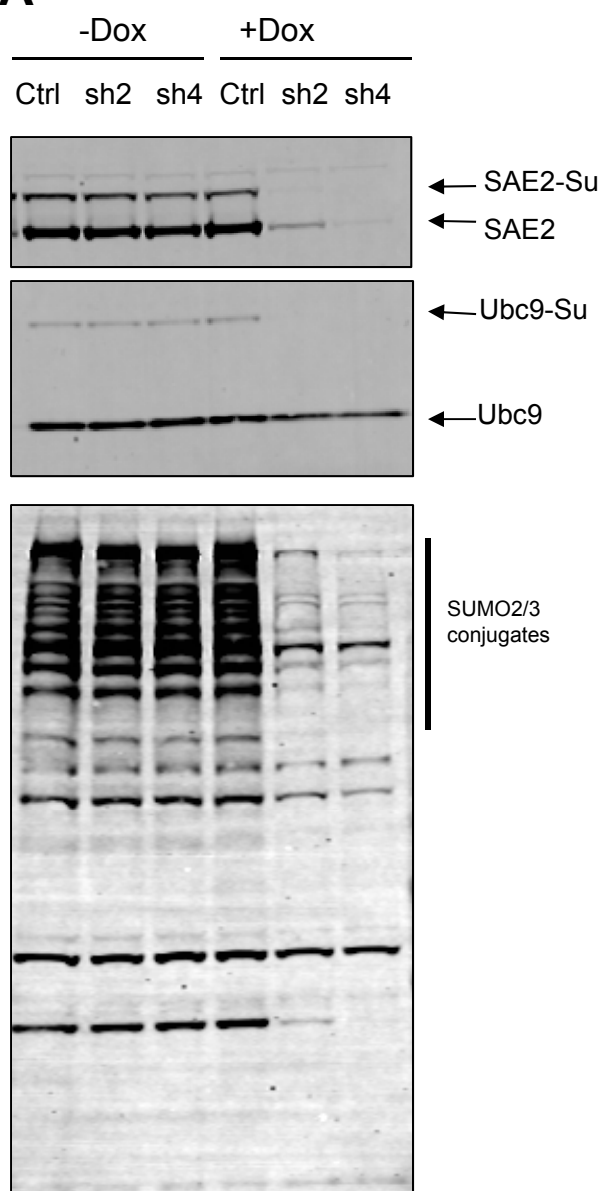

**B**

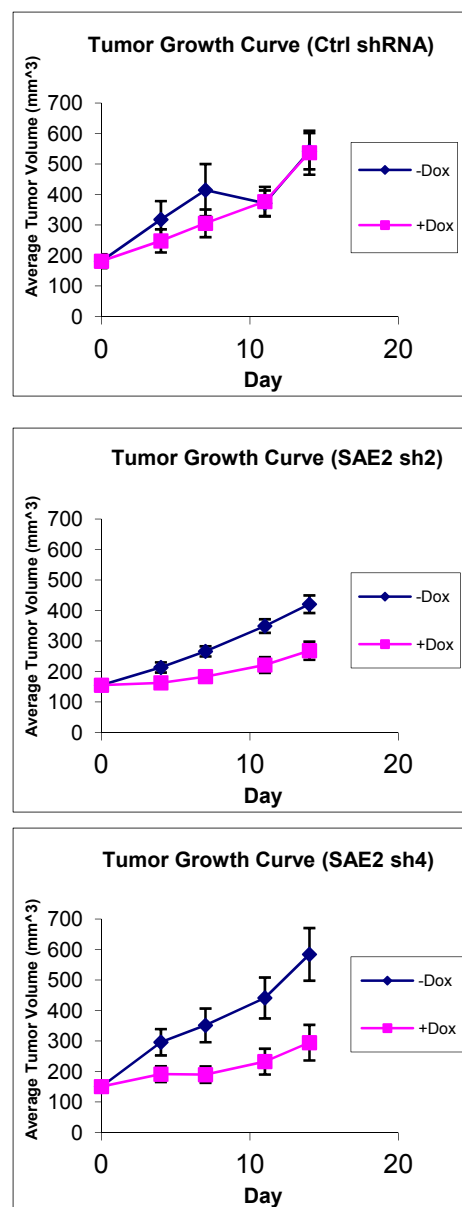

Supplement: S6 Fig — (A) HT29 cells were infected with Dox inducible SAE2 shRNAs (sh2 and sh4) or empty vector control (ctrl). Cells were treated with Dox for 5 days (+ Dox) or untreated (- Dox). Protein lystes were immunoblotted for SAE2, Ubc9, and SUMO2/3. (B) HT29 cells harboring tet-on ctrl sh or SAE2 sh2 and SAE2 sh4 were injected subcutaneously in immunocompromised mice. Dox treatment starts at D14 after injection (set as D0). (PDF) [file pone.0123882.s006.pdf]
